# A non-invasive strategy for suppressing asthmatic airway inflammation and remodeling: Inhalation of nebulized hypoxic hUCMSC-derived extracellular vesicles

**DOI:** 10.3389/fimmu.2023.1150971

**Published:** 2023-04-05

**Authors:** Xiaowei Xu, Ying Wang, Xinkai Luo, Xuerong Gao, Weifeng Gu, Yongbin Ma, Lili Xu, Mengzhu Yu, Xi Liu, Jiameng Liu, Xuefeng Wang, Tingting Zheng, Chaoming Mao, Liyang Dong

**Affiliations:** ^1^ Department of Nuclear Medicine, The Affiliated Hospital of Jiangsu University, Zhenjiang, Jiangsu, China; ^2^ Department of Respiratory Diseases, The Affiliated Huai’an Hospital of Xuzhou Medical University, Huai’an, Jiangsu, China; ^3^ Department of Central Laboratory, Jintan Hospital of Jiangsu University, Changzhou, Jiangsu, China; ^4^ Department of Respiratory Diseases, The Affiliated People’s Hospital of Jiangsu University, Zhenjiang, Jiangsu, China; ^5^ Department of Paidology, The Affiliated Hospital of Jiangsu University, Zhenjiang, Jiangsu, China; ^6^ Department of Central Laboratory, The Affiliated Hospital of Jiangsu University, Zhenjiang, Jiangsu, China

**Keywords:** inhalation device, nebulized administration, mesenchymal stem cells, extracellular vesicles, asthma, lung injury

## Abstract

Mesenchymal stromal cell-derived extracellular vesicles (MSC-EVs) are extremely promising nanoscale cell-free therapeutic agents. We previously identified that intravenous administration (IV) of human umbilical cord MSC-EVs (hUCMSC-EVs), especially hypoxic hUCMSC-EVs (Hypo-EVs), could suppress allergic airway inflammation and remodeling. Here, we further investigated the therapeutic effects of Hypo-EVs administration by atomizing inhalation (INH), which is a non-invasive and efficient drug delivery method for lung diseases. We found that nebulized Hypo-EVs produced by the atomization system (medical/household air compressor and nebulizer) maintained excellent structural integrity. Nebulized Dir-labeled Hypo-EVs inhaled by mice were mainly restricted to lungs. INH administration of Hypo-EVs significantly reduced the airway inflammatory infiltration, decreased the levels of IL-4, IL-5 and IL-13 in bronchoalveolar lavage fluid (BALF), declined the content of OVA-specific IgE in serum, attenuated the goblet cell metaplasia, and the expressions of subepithelial collagen-1 and α-smooth muscle actin (α-SMA). Notably, Hypo-EV INH administration was generally more potent than Hypo-EV IV in suppressing IL-13 levels and collagen-1 and α-SMA expressions. RNA sequencing revealed that various biological processes, such as cell adhesion, innate immune response, B cell activation, and extracellular space, were associated with the activity of Hypo-EV INH against asthma mice. In addition, Hypo-EVs could load exogenous miR-146a-5p (miR-146a-5p-EVs). Furthermore, INH administration of miR-146a-5p-EVs resulted in a significantly increased expression of miR-146a-5p mostly in lungs, and offered greater protection against the OVA-induced increase in airway inflammation, subepithelial collagen accumulation and myofibroblast compared with nebulized Hypo-EVs. Overall, nebulized Hypo-EVs effectively attenuated allergic airway inflammation and remodeling, potentially creating a non-invasive route for the use of MSC-EVs in asthma treatment.

## Introduction

Asthma is a chronic respiratory disease characterized by airway inflammation and airway remodeling (1). This disease affects more than 350 million people worldwide and has become a serious global public health concern (2). Current asthma therapy, including corticosteroids and long-acting β2-adrenoceptor agonists, focuses on symptom management rather than disease regression (3). Moreover, high doses of corticosteroids have side effects (4). New treatment strategies are still needed.

An increasing amount of evidence showed that exogenous mesenchymal stem cells (MSCs) effectively elicited inhibitory effects on airway inflammation and airway remodeling in asthmatic mice through the secretion of paracrine factors (5, 6). Extracellular vesicles (EVs) are nano-scale membrane vesicles released by almost all cell types (7). Transplantation of MSC-derived EVs (MSC-EVs) and MSC exhibits similar therapeutic effects on the alleviation of lung inflammation and reduction of collagen fiber content in chronic asthma mice (8), confirming MSC-EVs are a major kind of functional forms of MSCs (9). Strikingly, MSC-EVs possess conspicuous advantages over cell-therapy, such as high biosafety, low immunogenicity, easy storage, and can even be considered as an off-the-shelf product (10). Thus, MSC-EVs might represent an extremely promising cell-free therapeutic strategy for asthma, as confirmed in various experimental asthma models (11–14).

To data, most published articles on the use of MSC-EVs in the treatment of asthma (mice model) discussed their administration by tail vein. It should be noted that large amount of MSC-EVs administered intravenously (IV) will converge in the liver (15–19), which not only increased the metabolic burden of body but also caused the MSC-EV waste [especially the yield of MSC-EVs is insufficient today (20)]. Moreover, most patients with asthma might not accept this invasive administration route. The atomizing inhalation (INH) has been gaining immense attention in the treatment of lung damage, because it offers the advantages of rapid onset of action, reduced dosage amount, localized action, and avoidance of first-pass effect. We speculated that inhalation of nebulized MSC-EVs might be effective for the treatment of asthma, which has never been reported before.

In this study, we first created a nose-only and high-effective inhalation exposure system to mouse by simulating the process of human clinical atomization administration. We previously found that hypoxic environment (5% O_2_) could promote hUCMSCs to release more EVs (called Hypo-EVs), and these Hypo-EVs (IV administration) were generally more potent than normoxic hUCMSC-EVs (21% O_2_) in suppressing airway inflammation and remodeling in chronic asthmatic mice (14). Thus, we selected hypoxic hUCMSCs as the source of MSC-EVs and further explored the anti-asthma potential of Hypo-EVs when therapeutically INH administered to established disease pathology.

## Materials and methods

### Instrument, inhalation and mouse holding chambers

Medical/household compression atomizer, including air compressor (DM-YWH 01L) and nebulizer, was procured from Demi medical equipment Co.,ltd (Guangzhou, China). Inhalation chamber was made using silica glass, and consisted of 3 major parts (1) inlet pipe (height 5 cm, inner diameter 2cm); (2) six-way pipe (inner pipe:length 4cm, inner diameter 2cm; outer pipe: length 6 cm, inner diameter 3 cm; vent: diameter 0.4 cm); (3) base fixing device.

Centrifuge tubes (diameter 2.8 cm) routinely used in the laboratories were designed as mice holding chambers. The tips of the centrifuge tubes were removed to make a hole of around 0.9 cm diameter.

### Cell culture

HUCMSCs used in this study were generated from fresh umbilical cord samples as we reported previously (21), and maintained in stem cell culture medium (Cyagen, Guangzhou, China) at 37°C with 5% CO_2_. HUCMSCs between passages 3–7 were used for all experiments.

### Extraction and characterization of hypo-EVs

HUCMSCs were cultured in serum-free culture medium for 24 h under hypoxic (5% O_2_) conditions (Hypo-MSCs) (14). The cell supernatants were collected and centrifuged at 300×g for 10 min, 2000×g for 20 min to discard cell debris. Then, Hypo-EVs were isolated by ultracentrifugation (Beckman Coulter Optima L-100 XP ultracentrifuge, Miami, FL) at 100,000×g for 90 min as previously described (21). After that, the pellets were collected, washed, and resuspensed in PBS (Hypo-EVs solution). All centrifugations were performed at 4°C and the Hypo-EVs solution was stored at -80°C. For the isolation of Hypo-EVs engineered by miR-146a-5p (miR-146a-5p-EVs), Hypo-MSCs were transfected with 50 nM miR-146a-5p mimic or mimic NC (GenePharma, Shanghai, China) using Lipofectamine 2000 (Invitrogen, Carlsbad, CA), followed by culturing with conditioned medium and ultracentrifugation.

The protein concentration of EVs was determined by using BCA protein assay kit (Beyotime, Nantong, China). EV surface markers TSG101 (ab133586, Abcam, Cambridge, MA), and HSP70 (ab181606, Abcam) were detected by Western blot (WB) as our previous description (22). Shape and ultrastructure of EVs were observed by transmission electron microscopy (JEM-1200EX; JEOL Ltd., Tokyo, Japan). The particle size distribution of EVs was determined by nanoparticle trafficking analysis using ZetaView PMX 110 (Particel Metrix, Meerbusch, Germany) according to the manufacturer’s protocols.

### Nebulized hypo-EVs tracking in mice

Hypo-EVs were labeled with Dir (Invitrogen) as our previous report (17). BALB/c mouse was nebulized with Dir-labeled Hypo-EVs (40 μg diluted in 0.5 mL PBS). The mice were sacrificed at each time point (day 1 and 7) respectively post-administration. The ex vivo fluorescence images of brain, heart, liver, spleen, lung, kidney, stomach and intestines were visualized using Xtreme II (BRUKER, Bremen, Germany) according to the manufacturer’s protocol.

### Mouse model of chronic asthma with nebulized hypo-EVs

Six-week-old female BALB/c mice were purchased from the Comparative Medicine Centre of Jiangsu University (Zhenjiang, China). The OVA-induced chronic asthma model has been previously described by our team (14). Briefly, apart from the control group, the mice were sensitized on day 0, 7, and 14 with 40 μg OVA (Sigma, Poole, UK) and 2 mg 10% aluminum hydroxide (Sigma) in PBS by intraperitoneal injection. Then, the sensitized mice were challenged with aerosol OVA (5%) in a plastic chamber (30 × 20 × 15 cm) three times per week from days 21 to 53. Aerosol OVA particles were created from a compression atomizer (403 M; YUWELL, Zhenjiang, China), directed into the plastic chamber, and vented to a fume hood.

A therapeutic regimen was instigated by inhaling 40 μg Hypo-EVs (suspended in 0.5 mL PBS, EVs-INH group) on day 26. Mice from EVs-IV group were treated with Hypo-EVs (40 μg suspended in 0.1 mL PBS) by intravenous injection. After four times treatment (day 26, 33, 40 and 47), the mice were sacrificed on day 55.

### Analysis of cells and inflammatory cytokines in bronchoalveolar lavage fluid

The BALF was collected as described in our previous study (23), and centrifuged (1500 rpm for 5 min) to separate the cells and supernatants. Cell pellets were resuspended in PBS (1 mL), and total inflammatory cells was counted using Neubauer hemocytometer, and eosinophils count was performed using Wright and Giemsa staining (BASO, Zhuhai, China). The supernatants were kept at -80°C until they were used for cytokine analysis. The concentrations of IL-4 IL-5, and IL-13 in the BALF were determined by using commercial enzyme-linked immune sorbent assay (ELISA) kits (Multi Sciences, Hangzhou, China) following the manufacturer’s instructions. The absorbance of the final reactant was measured at 450 and 630 nm using an ELISA plate reader (BioTek, Biotek Winooski, Vermont).

### Measurement of serum OVA-specific IgE

The serum was prepared from mouse whole blood (mouse orbits). OVA-specific IgE in serum was measured using ELISA. Briefly, the 96-well plate was coated with 100 µL of OVA (100 µg/mL) per well and blocked with 5% skim milk. After washing, 1:250 dilution of goat anti-mouse IgE (Abcam) and HRP-conjugated rabbit anti-goat secondary IgG (1:5000, Multisciences) were used for detection. It was read at 450 nm in an ELISA plate reader (BioTek).

### Lung histopathology

Lung tissues were collected, fixed with 10% neutral buffered formalin (48 h) and embedded in paraffin fixation. Then, 4-μm thick sections (3 sections per animal) were cut and stained with hematoxylin and eosin (HE), periodic acid–Schiff (PAS), and Masson trichrome. All pictures were captured using a Nikon microscope (Tokyo, Japan). Peribronchial inflammation score (grades 0-4) was evaluated in a blind-way (24). Goblet cell hyperplasia (grades 0-4) was determined using the method described by Padrid et al. (25). Image-Pro Plus software (Version X; Adobe, San Jose, CA) was used to quantify the areas occupied by collagen (blue, Masson trichrome staining), which were subsequently divided by the total area examined (as the percentage of collagen fibers) (8). At least 6 bronchioles were counted in each slide, and then, the mean inflammation score, goblet cell hyperplasia score, and percentage of collagen fibers were calculated for each mouse.

### Immunohistochemistry

IHC were performed as described in previous study (26). Briefly, mouse lung tissue sections were incubated with an antibody collagen-1 (GB111364, diluted 1: 500; Servicebio, Whhan, China) or a-SMA (GB11022-3, 1:1000 dilution; Servicebio) overnight at 4°C, followed incubating by HPR-conjugated secondary antibody (GB23303 or GB23301, 1:1000 dilution; Servicebio). Diaminobenzidine was used as the substrate. Integrated optical density of collagen-1 and a-SMA were detected by using Image-Pro Plus software.

### RNA sequencing

Total RNA was extracted from OVA and EVs-INH mouse lungs using RNAiso Plus reagent (Takara Bio Inc., Japan). The RNA integrity was evaluated using Agilent Bioanalyzer 2100 (Agilent, Santa Clara, CA). RNA-Seq experiment was carried out by LC-BIO Bio Technology (Hangzhou, China). After sequencing, the data (|log FC|>1 and adjusted *P*< 0.05) were further analyzed using LC-Bio Cloud Platform (https://www.omicstudio.cn/) for heat map, and using DAVID Bioinformatics Tesources (https://david.ncifcrf.gov/home.js) for gene ontology enrichment.

### RNA isolation and quantitative real-time PCR

Total RNA was extracted using RNAiso Plus (Takara) or mirVana RNA isolation kit (Ambion, Austin, TX) according to the manufacturer’s manual. All of the primers for real-time PCR (Pank3, Zfp59, Arhgef9, Tubb1, Trpv4, Casc1, Gapdh, miR-146a-5p and U48) were purchased from Genecopoeia (Germantown, MD). Real-time PCR was performed using All-in-oneTM qPCR Mix (Genecopoeia) on a QuantStudio 5 Real-Time system (Thermo Fisher Scientific, Waltham, MA). Date was calculated by 2^−ΔΔCt^ method based on our previous description (27). The threshold cycle (CT) indicates the fractional cycle number at which the amount of amplified target reaches a fixed threshold. ΔCT (test) = CT (target, test) – CT (ref, test), ΔCT (calibrator) = CT (target, calibrator) – CT (ref, calibrator), ΔΔCT = ΔCT (test) – ΔCT (calibrator). The levels of mRNA and miR-146a-5p were normalized to Gapdh and U48 respectively.

### WB

WB analysis of total protein from the lung tissues was performed as previously described (22). Equal amounts of proteins (50 μg) were electrophoresed in 10% sodium dodecyl SDS-PAGE and transferred onto polyvinylidene difluoride (PDVF) membranes. After blocking in a nonfat milk solution, the PDVF membranes were incubated with primary antibodies specific for TRAF6 (E-AB-18251, Elabscience, Wuhan, China), TIRAP (ab17218, Abcam) and GAPDH (60,004–1-lg, Proteintech, Rosemont, IL) overnight. The membranes were then incubated with horseradish peroxidase-conjugated secondary antibodies (ab97051 or ab6728, Abcam) at room temperature for 1 h. Next, the PVDF membranes were incubated with enhanced chemiluminescence reagent (Merck Millipore, Billerica, MA) before detection using a ChemiScope series 4300 (CLINX Science Instruments, Shanghai, China).

### Safety assessment of nebulized hypo-EVs

Mice were nebulized with Hypo-EVs (40 μg diluted in 0.5 mL PBS) at day 0, 7, 14, 21, 28, 35, 42, and 49 (total eight times, 40 μg/time). Healthy mice were used as controls. Mice survival and weight was recorded once a week. At 56 days, mice serum was collected and the serum biochemistry, including alanine aminotransferase (ALT), aspartate aminotransferase (AST), urea nitrogen (UREA) and creatinine (CREA), were detected using Beckman Coulter AU2700 automatic biochemical analyzer (Beckman). After mice were sacrificed, the major organs (heart, liver, spleen, lung, kidney, stomach, and intestines) were harvested for HE staining to assess the histological changes.

### Statistical analysis

The statistical analyses were performed with GraphPad Prism (Version 5.0; La Jolla, CA). Data are expressed as mean ± SD. The groups were compared using the one-way analysis of variance (Tukey Kramer *post hoc* tests) or Student’s t-test. A value of *P* < 0.05 was considered significant.

## Results

### Design of inhalation device for mice

**Figure 1:**
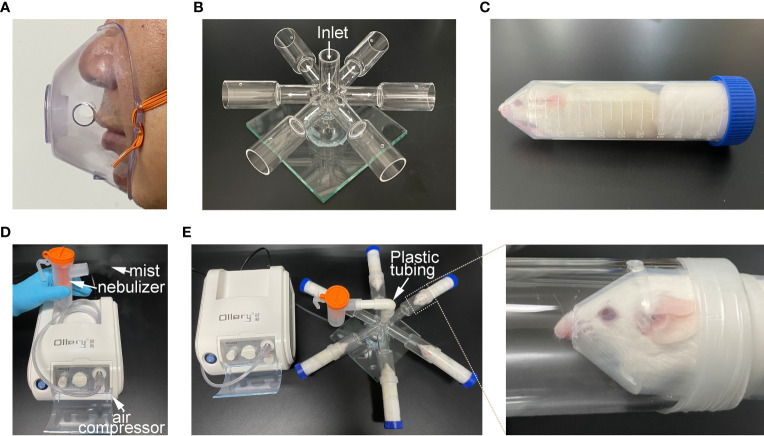
Construction of atomization inhalation system for mice. **(A)** atomization inhalation mask commonly used by human beings. **(B)** Six-way pipe inhalation chamber for mice depicting the flow pattern of the aerosol. This chamber consisted of three parts (inlet pipe, six-way pipe and base fixing device). **(C)** Holding chamber for mouse. **(D)** medical/household air compressor and nebulizer set-up. **(E)** Working diagram of mouse atomization inhalation system.

To better simulate the process of human clinical atomization administration, we first created an aerosol inhalation device for mice by imitating the human atomization mask (nose-only exposure, **Figure 1A**). This device includes an inhalation chamber and a mouse-holding chamber. The inhalation chamber has a six-way pipe with small cylindrical vial, and a small hole was made in the wall of each cylindrical vial, in order to minimize any pressure buildup inside the aerosol chamber. Six-furcations of the inhalation chamber were placed at the equidistant levels from the inlet to ensure uniformity in the dose delivered to each mouthpiece and ultimately to the delivery ports of the animal restrainers (**Figure 1B**). Centrifuge tubes (50 mL) were used as the mouse-holding chambers. The animals were restrained with the medical cotton ball, which could prevent any plausible change in the direction of the mouse’s movement. In addition, the tips of the centrifuge tubes were removed to make a smooth edge hole, so that nose of the mouse can be easily inserted (**Figure 1C**).

The medical air compressor attached to the nebulizer provides a positive pressure for the generation of aerosol mist (**Figure 1D**). Hypo-EVs were placed in the nebulizer. Plastic tubing was employed to connect the mouth of the nebulizer with the inlet pipe of inhalation chamber. Thus, the mist generated from the nebulizer could be transfer to the delivery port and be inhaled by the mouse restricted in the mouse-holding chamber (**Figure 1E**).

### Identification of nebulized hypo-EVs

**Figure 2:**
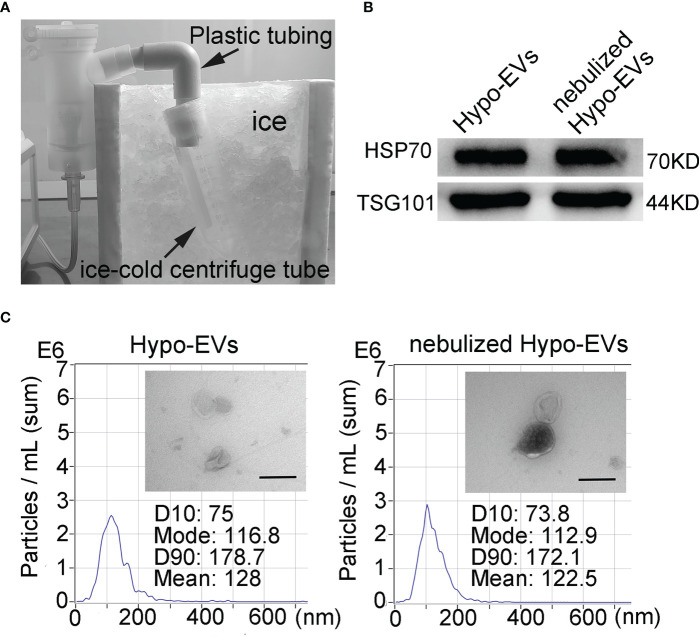
Characterization of Hypo-EVs. **(A)** The flowchart shows the process that nebulized Hypo-EVs ejected from nebulizer were liquefied by using an ice-cold centrifuge tube. **(B)** Western blot analysis of TSG101 and HSP70 expression in nebulized Hypo-EVs and fresh extracted Hypo-EVs. **(C)** EVs were observed under a transmission electron microscope (Scale bars = 200 nm), the images are shown at ×60000, and the size distributions were measured using the Nanoparticle Tracking Analysis.

To allow the analysis of the characteristics of nebulized Hypo-EVs, nebulized Hypo-EVs ejected from nebulizer were liquefied by using an ice-cold centrifuge tube (**Figure 2A**). Subsequently, the collected liquid containing Hypo-EVs were subjected to ultracentrifugation. Then, the deposit was examined by using WB, nanoparticle tracking analysis (NTA), and transmission electron microscope (TEM). Hypo-EVs freshly extracted from hypoxic hUCMSC culture medium were used as control. WB revealed that several EV markers including tumor susceptibility gene 101 (TSG101) and heat shock protein 70 (HSP70) (28) were detected in these EVs (**Figure 2B**). NTA exhibited that the mean sizes of Hypo-EVs and nebulized Hypo-EVs were 128 and 122.5 nm, respectively (**Figure 2C**). TEM showed that nebulized Hypo-EVs and Hypo-EVs were the same in terms of the round nanoparticles and complete membranous structure (**Figure 2C**), indicating that nebulized Hypo-EVs produced by the atomization system maintain excellent structural integrity.

### The biodistribution of nebulized hypo-EVs in mice

**Figure 3:**
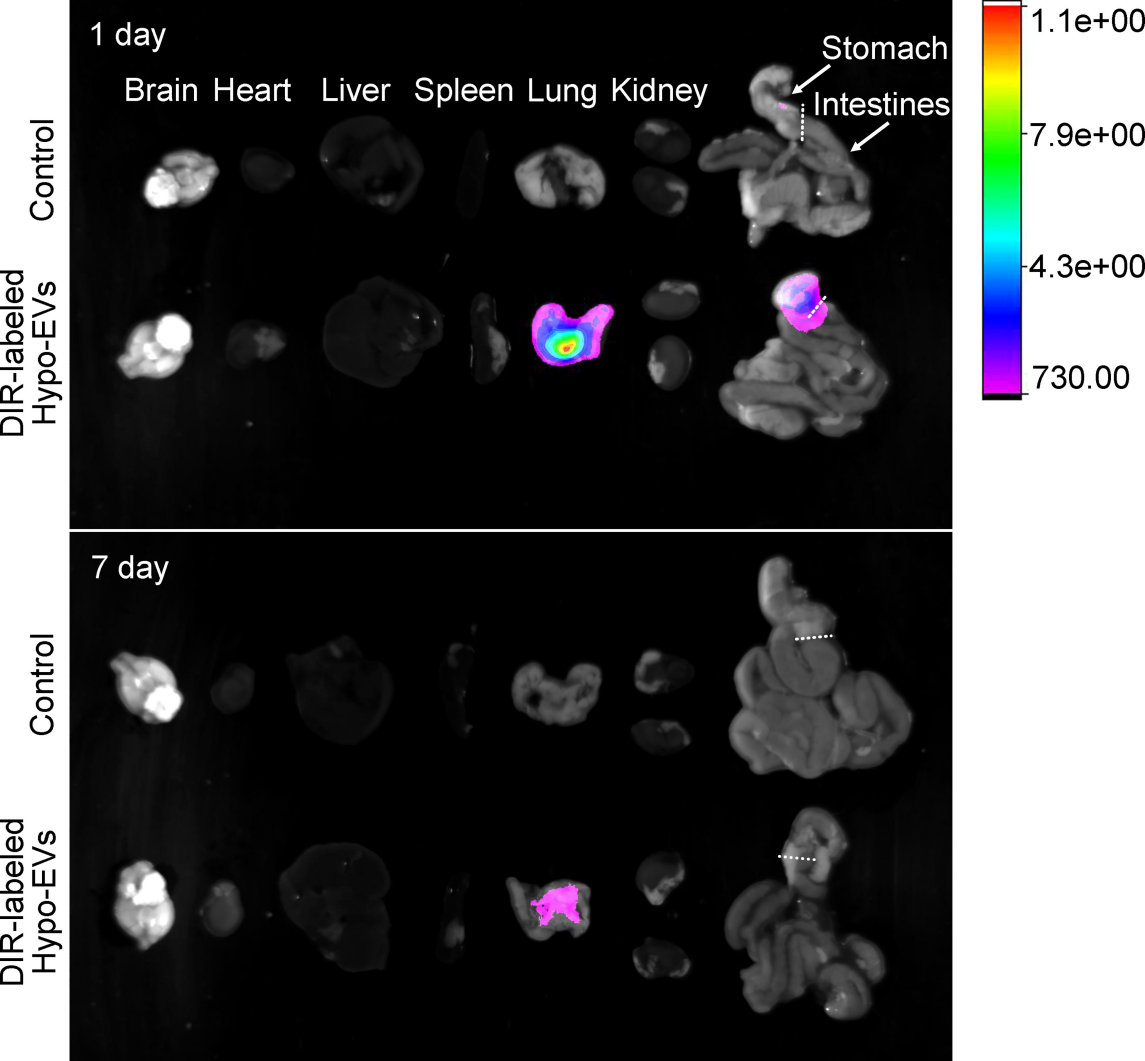
Biodistribution of Dir-labeled Hypo-EVs in major organs (brain, heart, liver, spleen, lung, kidney, stomach, and intestines) was visualized at 1 and 7 day after nebulized inhalation administration.

To determine the biodistribution of nebulized Hypo-EVs in BABL/C mice, Hypo-EVs were labeled with DiR first. Then, the DiR-labeled Hypo-EVs were administered through the nebulized route, and subsequently the organs of mice were dissected and the fluorescence intensity was detected. Mice synchronously inhaled PBS as a control. As shown in **Figure 3**, at 1 day after the DiR-labeled Hypo-EV inhalation, the strongest fluorescence intensity was observed in the lungs, with low intensity in the stomach and no accumulation in the brain, heart, liver, spleen, kidney, or intestines. At 7 day after nebulization, the fluorescence intensity was only enriched in the lungs, and decreased compared with 1 day.

### Inhalation of nebulized hypo-EVs attenuated OVA-induced chronic airway inflammation in mice

**Figure 4:**
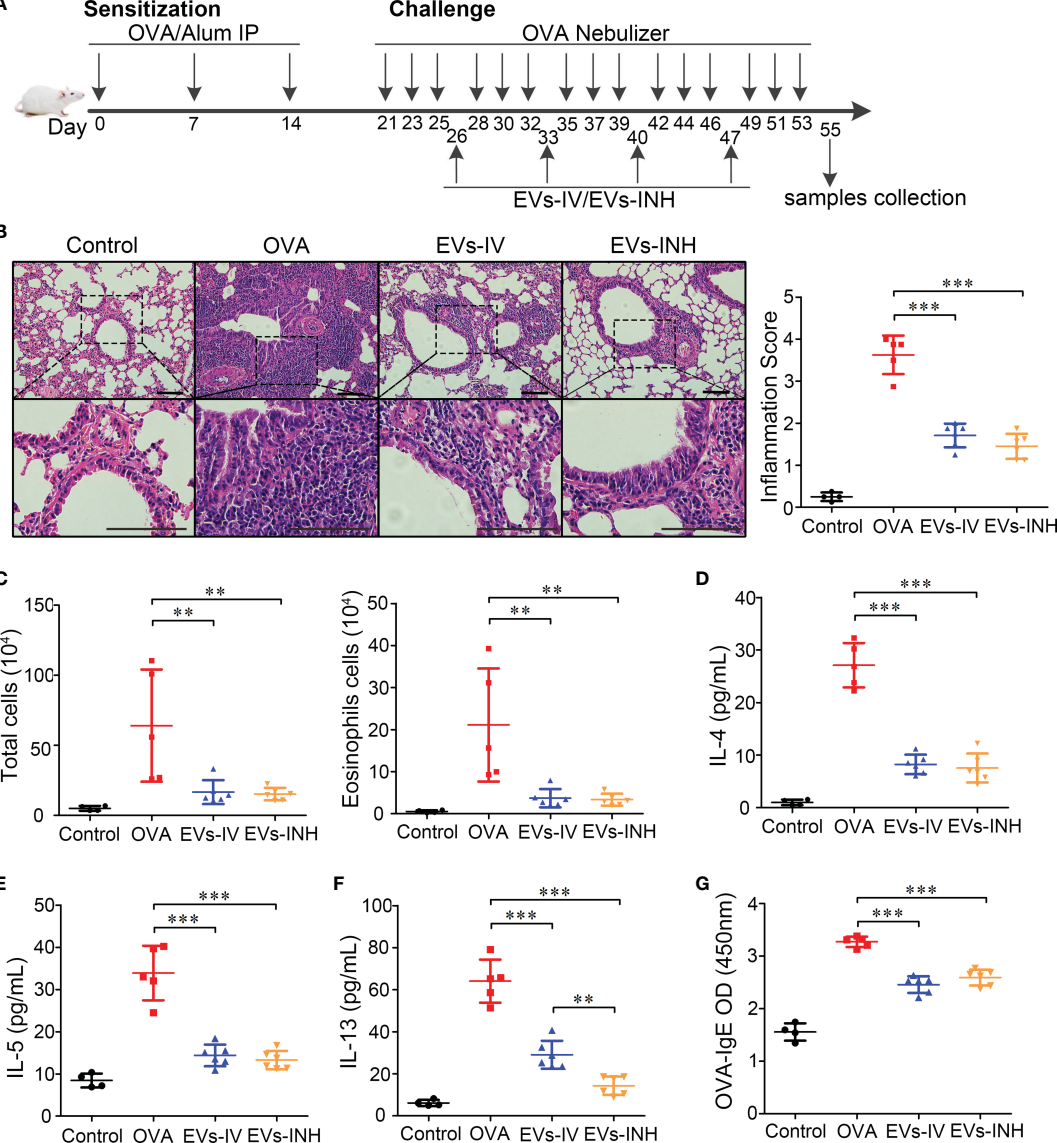
Inhalation of nebulized Hypo-EVs attenuated OVA-induced chronic airway inflammation in mice. **(A)** Experimental protocol for the development of chronic allergic asthma and treatment with Hypo-EVs. **(B)** Representative photographs of HE stained lung sections from each group (black bar =100 μm), the images are shown at ×200 (up panel) and ×400 (down panel), and the inflammatory infiltration was quantified by inflammation score. **(C)** Statistical analysis of the total inflammatory cells and eosinophils in the BALF. **(D–F)** IL-4, IL-5 and IL-13 levels in the BALF were measured by using ELISA. **(G)** The levels of OVA-specific IgE levels in serum were analyzed using ELISA. EVs-INH, Hypo-EV nebulization inhalation; EVs-IV, Hypo-EV intravenous injection. Each dot represents data from one animal and n = 4-6 per group. One-way analysis of variance (Tukey Kramer *post hoc* tests): **P < 0.01, ***P < 0.001.

To investigate the possible anti-inflammatory effects of nebulized Hypo-EVs on allergic airway reactivity, asthmatic mice were established, and the nebulized Hypo-EVs were inhaled (EVs-INH). Meanwhile, intravenous injection of Hypo-EVs (EVs-IV) was used as a positive control according to our previous report (14). The treatment regimen is illustrated in **Figure 4A**.

Lung histopathologic staining using HE showed that the asthmatic mice (OVA group) presented abundant infiltrates of peribronchial inflammatory cells compared with the control group, which was further identified by the increased inflammatory scores. Compared with OVA group, EVs-INH treatment significantly reduced the peribronchial inflammatory cell infiltration (**Figure 4B**). In addition, a differential cell count of bronchoalveolar lavage fluid (BALF) showed a significant decrease in total cells and eosinophil infiltrates in EVs-INH-treated mice (**Figure 4C**). Then, the type-2 cytokines IL-4, IL-5 and IL-13 in the BALF were determined. Our results showed that EVs-INH treatment dramatically decreased the protein levels of these three cytokines (**Figures 4D–F**). Compared with the EVs-IV gourp, significant lower levels of IL-13 were observed in EVs-INH-treated mice (**Figure 4F**). Serum levels of OVA-specific IgE were determined using ELISA, and as shown in **Figure 4G**, marked elevation of OVA-specific IgE were observed in OVA mice, which strongly decreased with EVs-INH treatment. Taken together, these findings implied that inhalation of nebulized Hypo-EVs attenuated chronic airway inflammation and suppressed Type-2 predominant immune activity in the OVA-induced murine asthmatic model.

### Inhalation of nebulized hypo-EVs prevented airway remodeling in chronic OVA mice

**Figure 5:**
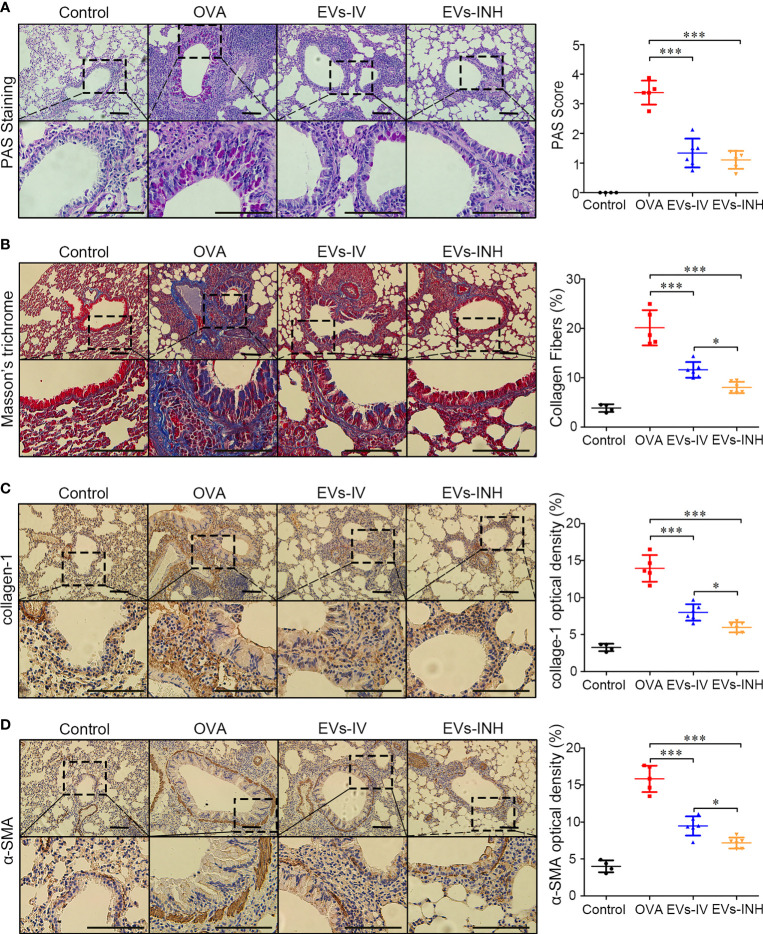
EVs-INH treatment prevented the airway remodeling in chronic asthma mice. **(A)** periodic acid-schiff (PAS) stained lung sections from each group (black bar = 100 μm), the images are shown at ×200 (up panel) and ×400 (down panel), and the goblet cell hyperplasia was quantified by PAS score. **(B)** Representative photomicrographs of airway stained with Masson trichrome (black bar = 100 μm), the images are shown at ×200 (up panel) and ×400 (down panel), and the percentage of collagen fiber content in airway was measured. Collagen-1 **(C)** and α-SMA **(D)** levels in airway were determined by immunohistochemistry staining, the images are shown at ×200 (up panel) and ×400 (down panel), and the percentage of immunostained area was quantified. Each dot represents data from one animal and n = 4-6 per group. One-way analysis of variance (Tukey Kramer *post hoc* tests): *P < 0.05, ***P < 0.001.

We further evaluated goblet cell hyperplasia in mouse lung tissues by PAS staining. As shown in **Figure 5A**, compared with control group, goblet cell numbers were significantly elevated in OVA-treated mice. EVs-INH treatment significantly reduced the aberrant OVA-induced promotion of goblet cell numbers. The subepithelial collagen deposition was investigated by using Masson trichrome and collagen-1 Immunohistochemical staining (IHC). As shown in **Figures 5B, C**, compared with OVA group or even EVs-IV group, less airway collagen fiber content or collagen-1 expression were observed in the lung tissues from EVs-INH-treated mice. The expression of α-SMA in lung tissues was further investigated as a detection of myofibroblast by using IHC. We found that EVs-INH inhibited the expression of α-SMA compared with that in the OVA or EVs-IV group (**Figure 5D**). These data suggested that EVs-INH treatment was effective in preventing airway remodeling in chronic asthma mouse.

### Identification of genes regulated by EVs-INH in chronic OVA mouse lung

**Figure 6:**
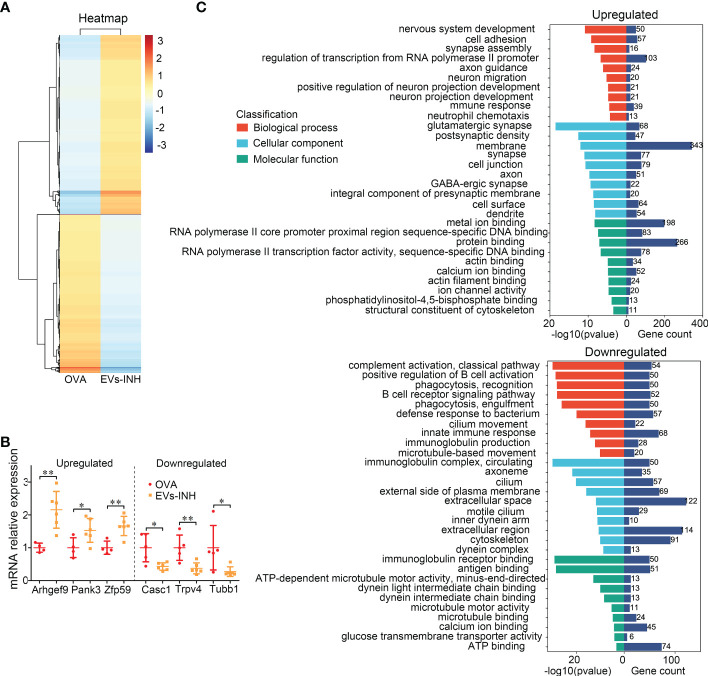
The differential expressed (DE) mRNAs in lungs between EVs-INH-treated mice and OVA-induced asthma mice. **(A)** Heat-map was constructed for DE miRNAs in EVs-INH vs OVA. **(B)** Real-time PCR verification of several of the lowlist differentially expressed genes. **(C)** GO analysis of the differentially up and down expressed genes in lungs between EVs-INH-treated mice and OVA-induced asthma mice. The top-ten statistically signifificant results identifified including biological process (BP), cellular component (CC), and molecular function (MF) are listed. Each dot represents data from one animal and n = 4-6 per group. Student’s t-test: *P < 0.05, **P < 0.01.

To better understand the molecular processes of nebulized Hypo-EVs, a global RNA sequencing of mouse lung from the OVA and EVs-INH groups was performed. Data were analyzed using conventional approaches based on Fragments Per Kilobase per Million mapped reads (FPKM), and the comparison generated a heat map of differentially expressed genes (|log FC| > 1 and adjusted *P*-value < 0.05, **Figure 6A**). Compared with OVA group, EVs-INH mice presented 969 upregulated mRNAs and 856 downregulated mRNAs (**Additional file 1**), which were validated by real-time PCR through the determination of the expression of the three most lowly overexpressed (*Pank3*, *Zfp59* and *Arhgef9*) or downregulated (*Tubb1*, *Trpv4* and *Casc1*) genes (**Figure 6B**). Then, respective gene ontology enrichment analysis was conducted using DAVID bioinformatics resources. To increase the confidence levels of the analyses, we only presented the results of the 10 most relevant terms for the analyses of biological process (BP), cellular component (CC), and molecular function (MF) (**Figure 6C** and **Additional file 2**).

BP showed that upregulated mRNAs were largely involved in regulation of transcription form RNA polymerase II promoter (11.3%), cell adhesion (6.3%), and nervous system development (5.5%). The downregulated mRNAs were involved in innate immune response (8.2%), defense response to bacterium (6.9%), complement activation (6.5%), and positive regulation B cell activation (6.1%).

CC revealed that most upregulated mRNAs were involved in membrane (37.7%), cell junction (8.7%), and synapse (8.5%). Downregulated mRNAs were involved in extracellular space (14.8%), extracellular region (13.8%), and cytoskeleton (11%).

MF analysis suggested that the upregulated mRNAs showed extensive binding capacities to many components, such as, protein binding (29.2%) and metal ion binding (21.7%). Downregulated mRNAs were involved in ATP binding (9%), antigen binding (6.2%) and immunoglobulin receptor binding (6.1%).

### Aerosol delivery of hypo-EVs carrying miR-146a-5p (miR-146a-5p-EVs) more inhibited airway inflammation and remodeling in chronic asthma mice

**Figure 7:**
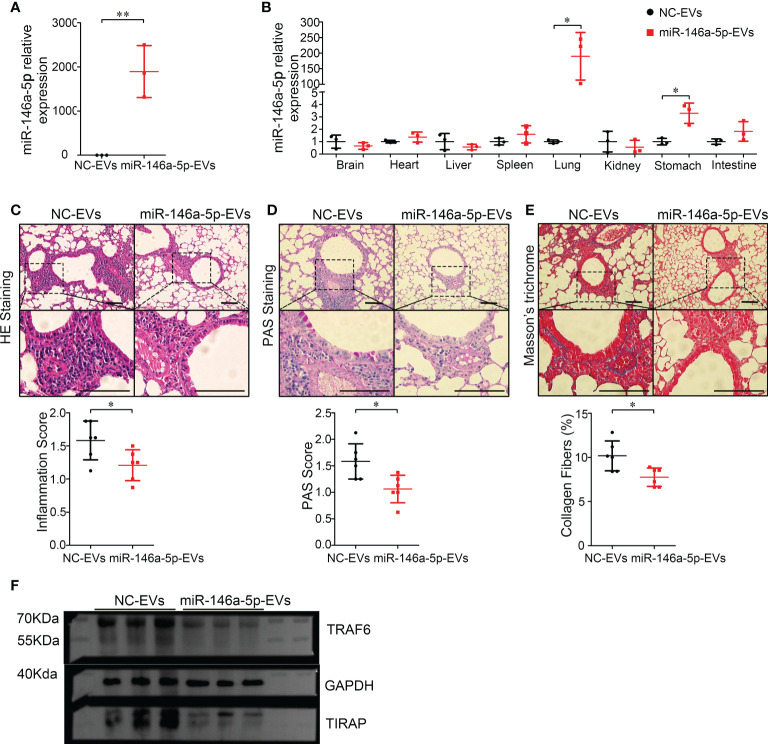
Inhalation of Hypo-EVs carrying miR-146a-5p (miR-146a-5p-EVs) more inhibited airway inflammation and remodeling in chronic asthma mice. **(A)** Relative miR-146a-5p levels in miR-146a-5p-EVs and NC-EVs were detected by real-time PCR (n=3). **(B)** Levels of miR-146a-5p in major organs (brain, heart, liver, spleen, lung, kidney, stomach, and intestines) following administration by aerosol miR-146a-5p-EVs and NC-EVs (n=3). Representative photographs of HE **(C)**, PAS **(D)**, and Masson trichrome **(E)** stained lung sections from each group (black bar = 100 μm), the images are shown at ×200 (up panel) and ×400 (down panel), and respectively inflammatory score, PAS score and and the percentage of collagen fiber content in airway were quantified (n=6). **(F)** The protein levels of TRAF6 and TIRAP in the lung samples were analyzed by western blot, and the GAPDH was as a loading control (n=3). Each dot represents data from one animal. Student’s t-test: *P < 0.05, **P < 0.01.

Recently, MSC-EVs are not only regarded as a next generation cell-free therapeutic tool (29), but also as a nano-scale gene delivery platform, especially miRNAs (30, 31). MiRNAs are potential candidates for asthma therapy. For example, miR-146a-5p was reported to efficiently protect mice against OVA-induced allergic asthma (32, 33), and also modulate anti-fibrosis responses (34). Thus, the anti-asthma effects of miR-146a-5p-EVs (Hypo-EVs carrying miR-146a-5p) might be more profound than those of Hypo-EVs. As shown in **Figure 7A**, compared with NC-EVs (Hypo-EVs carrying miR-146a-5p mimic control), a significant increase in miR-146a-5p levels was observed in miR-146a-5p-EVs. The accumulation of nebulized Hypo-EVs-delivered miR-146a-5p in the asthma mice was investigated. Compared with nebulized NC-EVs, nebulized miR-146a-5p-EVs resulted in significantly higher levels of miR-146a-5p in the lungs (190 fold) and stomach (3.3 fold), but not in brain, heart, liver, spleen, kidney, and intestines (**Figure 7B**). Notably, miR-146a-5p-EV treatment exhibited more inhibitory effects on airway inflammation (**Figure 7C**), goblet cell hyperplasia (**Figure 7D**), and collagen fiber content (**Figure 7E**) in chronic asthma mice. In addition, the downstream effector of miR-146a-5p, including TNF receptor-associated factor 6 (TRAF6) (35) and TIR domain-containing adaptor protein (TIRAP) (36), which are related to airway epithelial cell injury and airway inflammatory response in asthma, were decreased (**Figure 7F**).

### Toxicity of inhalation of nebulized hypo-EVs

**Figure 8:**
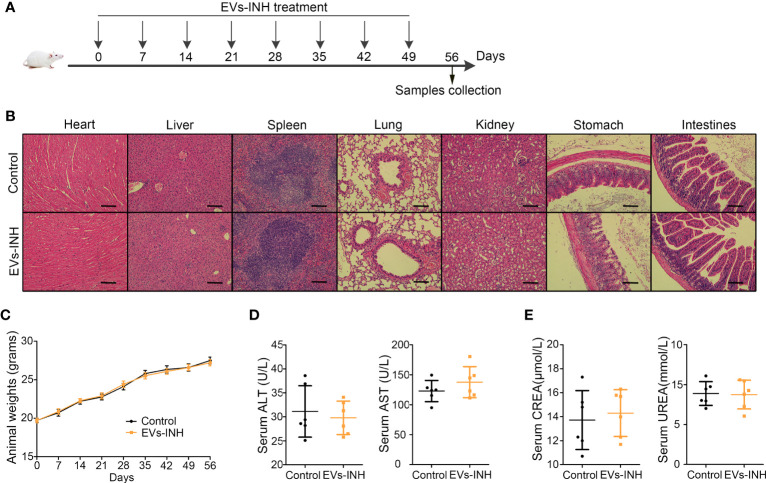
Biocompatibility evaluation of EVs-INH (n=6). **(A)** Schematic diagram for EVs-INH treatment. **(B)** HE staining, scale bar=100 μm, and the images are shown at ×200. **(C)** Mouse body weights. Indicators reflected the physiological function of the **(D)** liver (ALT and AST) and **(E)** kidney (CREA and UREA) were detected. ALT, glutamic pyruvic transaminase; AST, glutamic oxaloacetic transaminase; CREA, Creatinine. Each dot represents data from one animal and n = 6 per group. Student’s t-test.

The safety of nebulized Hypo-EV treatment was examined during *in vivo* studies (**Figure 8A**). No animal died in 56 days (date not shown). Compared with the health mice (Control group), no obvious tissue damages and inflammatory infiltration happened after EVs-INH treatment in the HE staining of major organs (heart, liver, spleen, lung, kidney, stomach, and intestines) (**Figure 8B**). EVs-INH had no impact on mouse body weight (**Figure 8C**). The liver and kidney function-related blood biochemical values (ALT, AST, UREA, and CREA) in the serum of mice on Day 56 were assayed. Compared with the healthy mice, no significant differences have been found in the mice after EVs-INH treatment (**Figures 8D, E**). All these results indicated that the EVs-INH possessed excellent biosafety.

## Discussion

In recent years, MSC-EVs, a kind of nano-scale membranous vesicles secreted by MSC, are considered to be a best and market-promising substitute for MSC, science they are better defined, less complicated, easy storage, tiny, and seedless (9, 10). Numerous studies confirmed that intravenous administration (IV) of MSC-EVs exerted therapeutic potential to respiratory diseases in animal models (37), while, the investigation of nebulized inhalation (INH), a superior no-invasive drug delivery method for the treatment of pulmonary disease, are precious few. Dinh et al. (38) used air compressor and nebulizer to produce nebulized human bone marrow-derived MSC-EVs and presented a series of studies utilizing these EVs through the whole-body exposure to rat in a box to treat different models of lung injury and fibrosis. Shi et al. (39) used vibrating mesh nebulizers to produce nebulized human adipose-derived MSC-EVs (hADMSC-EVs). Then they placed the mouse head into the nebulizer nozzle and investigated the effect of hADMSC-EVs in the P. aeruginosa-induced murine lung injury. Zhao et al. (40) used commercially available rodent inhalers to produce nebulized hUCMSC-EVs. After intratracheal administration, the therapeutic effects of these EVs on lipopolysaccharide (LPS)-induce animal models were explored. In this study, we used medical/household air compressor and nebulizer to produce nebulized Hypo-EVs owing to their low prices, wide application, tiny smoke particles (1-5 μm), and adjustable fog. The atomized Hypo-EVs could still maintain excellent structural integrity, which further verified the feasibility of MSC-EV INH administration. More importantly, we created an available mouse inhalation device that consisted of six-way mouse inhalation chamber and mouse-holding chamber. Compared with the above mentioned animal inhalation device, our device separately (1) simulated the atomization inhalation process of human beings and realized the nose-only EV exposure; (2) could be administered to multiple mice in parallel; (3) was cost-effective, which is particularly practical for scientists working in low-income countries. In short, we established a feasible, nose-only, high-efficient, and cheap atomization system for mice, which might promote the research progress of preclinical testing of MSC-EVs (or other EVs) administered through INH at laboratory scale.

Biodistribution of Dir-labelled Hypo-EVs in mouse major organs after INH administration revealed that fluorescence intensity mostly accumulated in the lungs. A low intensity was detected within the stomach at 1 day, which might be explained by the possibility of EVs being swallowed during the nebulization process, and this result was consistent with Shi’s report (39). Generally, INH route has the potential for lung targeting and avoids the trapping of EVs in the liver, which is commonly reported during IV administration (15–19).

Asthma is a very common Type-2 immune mediated chronic respiratory disease that lacks effective treatment strategy. Here, we found that Hypo-EVs INH treatment significantly decreased airway inflammatory cell infiltration, numbers of total and eosinophils cells, protein levels of Type-2 inflammatory mediators (IL-4, IL-5, IL-13), and OVA-specific IgE level. It also prevented airway remodeling, concomitant with the reduced number of goblet cell metaplasia, content of subepithelial collagen, and expressions of collagen-1 and pro-fibrogenic markers α-SMA. More importantly, the direct delivery of Hypo-EVs into the lungs *via* the INH route offered greater protection against the OVA-induced increase in IL-13 levels, subepithelial collagen and myofibroblast accumulation compared with IV delivery of these EVs. Obviously, INH route might be a more effective delivery strategy than IV for the treatment of asthma using MSC-EVs (such as Hypo-EVs).

MSC-EVs participate in many biological processes, such as tissue regeneration (41), immune responses (42), and anti-fibrosis (43). However, little is known about their regulatory role in asthma, especially when administered *via* INH. In this study, we investigated the lung mRNA profile in OVA-induced and EVs-INH-treated mice through RNA-Seq, and the reliability of the RNA-seq results was later confirmed by real-time PCR. GO analysis revealed that EVs-INH showed positive regulation on cell adhesion, nervous system development, cell junction, and so on; while had negative regulation on innate immune response, positive regulation B cell activation, extracellular space, extracellular region, and antigen binding, which indicated that EVs-INH might have multiple targets and multiple effects in asthma.

In this study, we packed miR-146a-5p, a famous anti-inflammatory and anti-fibrotic miRNA (32–34), into Hypo-EVs (miR-146a-5p-EVs) and further investigated the therapeutic effect of miR-146a-5p-EVs on asthma mice. INH administration of miR-146a-5p-EVs resulted in significantly higher levels of miR-146a-5p in the lungs, little in stomach, no in other tissues, indicating that miR-146a-5p-EV INH treatment could deliver miR-146a-5p relatively specifically to mouse lungs. Although, miRNAs are potential candidates for treating respiratory diseases, including asthma (44), an efficient delivery system to directly deliver them to lung still lack. Thus, INH administration of MSC-EVs-carrying miRNAs might provide a good reference. More importantly, we found that miR-146a-5p-EVs were generally more potent than Hypo-EVs in suppressing airway inflammation and remodeling in asthmatic mice, which further supported the notion that engineering MSC-EVs is an effective way to improve the therapeutic effect of MSC-EVs (45, 46).

In present study, we first investigated the effects of Hypo-EV INH administration on health mice. Hypo-EV INH treatment had no impact on mouse survival, body weight, pathology of major organs, liver and kidney functions, partly confirming that INH route was safe.

This study had a number of limitations. (1) We only used a single dosage of Hypo-EVs in our animal model. (2) The specific target cells and molecular mechanism of EVs-INH were not further explored. (3) Due to the lack of relevant equipment, airway hyperresponsiveness, another main component to asthma, was not detected. (4) The effectiveness and the safety of normoxic MSC-EVs on asthmatic or normal mice were not investigated.

In conclusions, we created a nose-only, high-efficient, and cheap inhalation device for rodents and confirmed that inhalation of nebulized Hypo-EVs could effectively reduce airway inflammation and reverse markers of airway remodeling in asthma mice. These findings provided ideas for the determination of the equipment to use in the atomization research of MSC-EVs and a noninvasive strategy for ameliorating asthma.

## Data availability statement

The datasets presented in this study can be found in online repositories. The name of the repository and accession number(s) can be found below: NCBI Gene Expression Omnibus; accession number GSE226639.

## Ethics statement

Human umbilical cord samples were obtained from informed, consenting mothers at Affiliated Hospital of Jiangsu University, and the study was approved by the Ethics Committee of Affiliated Hospital of Jiangsu University. The animal study was reviewed and approved by Institutional Animal Care and Use Committee of Jiangsu University.

## Author contributions

XX, YW, XinL, CM, TZ, LD conceived and designed the experiments. XX, YW, XinL, XG, YM, LX, MY, LD analyzed the data. XX, YW, XinL, XG, WG, XiL, JL, XW, LD performed the experiments. The manuscript was written by TZ, CM, LD. All authors contributed to the article and approved the submitted version.

## Supplementary material

Additional file 1 The DE mRNAs in lungs between EVs-INH-treated mice and OVA-induced asthma mice (|log FC| > 1 and adjusted *P*-value < 0.05).

Additional file 2 The top-ten statistically signifificant results of biological process (BP), cellular component (CC), and molecular function (MF).
